# High-level expression of Rad51 is an independent prognostic marker of survival in non-small-cell lung cancer patients

**DOI:** 10.1038/sj.bjc.6602665

**Published:** 2005-06-14

**Authors:** G-B Qiao, Y-L Wu, X-N Yang, W-Z Zhong, D Xie, X-Y Guan, D Fischer, H-C Kolberg, S Kruger, H-W Stuerzbecher

**Affiliations:** 1 Lung Cancer Research Institute & Cancer Center, Guangdong Provincial People's Hospital, Guangzhou, P.R. China; 2 Department of Clinical Oncology, the University of Hong Kong, P.R. China; 3 Clinic of Gynaecology and Obstetrics, University Clinic Schleswig-Holstein (UK S-H), Campus Luebeck, Ratzeburger Allee 160, D-23538 Lübeck, Germany; 4 Institute of Pathology, University Clinic Schleswig-Holstein (UK S-H), Campus Luebeck, Ratzeburger Allee 160, D-23538 Lübeck, Germany

**Keywords:** non-small-cell lung carcinoma, prognosis, tissue microarray, Rad51

## Abstract

High-level expression of Rad51, a key factor in homologous recombination, has been observed in a variety of human malignancies. This study was aimed to evaluate Rad51 expression to serve as prognostic marker in non-small-cell lung cancer (NSCLC). A total of 383 non-small-cell lung tumours were analysed immunohistochemically on NSCLC tissue microarrays. High-level Rad51 expression was observed in 29.4% (100 out of 340) of cases. Patients whose tumours displayed high-level Rad51 expression showed a significantly shorter median survival time of 19 *vs* 68 months (*P*<0.0001, log-rank test). Similarly T status, N status, M status, clinical stage and histological tumour grade were significant prognostic markers in univariate Cox survival analysis. Importantly, Rad51 expression (*P*<0.0001) together with tumour differentiation (*P*<0.009), clinical stage (*P=*0.004) and N status (*P=*0.0001) proved to be independent prognostic parameters in multivariate analysis. Rad51 expression predicted the outcome of squamous cell cancer as well as adenocarcinoma of the lung. Our results suggest that Rad51 expression provides additional prognostic information for surgically treated NSCLC patients. We hypothesise that the decreased survival of NSCLC patients with high-level expression of Rad51 is related to an enhanced propensity of tumour cells for survival, antiapoptosis and chemo-/radioresistance.

Lung cancer continues to be the most frequent cancer with approximately one million people worldwide dying of this disease each year (Carney, 2002). Non-small-cell lung cancer (NSCLC), which accounts for approximately 80% of all lung cancers, is conceptualised as a group of heterogeneous clinical entities that share a common cellular origin but have different clinical behaviours, and hence, different prognoses. Although surgery is the best therapeutic modality for patients with early stages of NSCLC, patients with the same clinical stage of NSCLC display considerable variability in recurrence and survival. Even after radical surgery, a significant proportion of patients may suffer from regional or distant recurrence (Buccheri and Ferrigno, 1994). Therefore, there is an urgent need for new parameters that can distinguish between patients with unfavourable prognosis and others with better prognosis. If individuals with poor prognosis could be identified at the time of surgery, their survival might be prolonged by more effective adjuvant therapy.

Rad51, one of the key factors of DNA repair by homologous recombination, has been shown to have antiapoptotic activity in tumour cells (Henning and Stuerzbecher, 2003). A large body of evidence indicates that Rad51 expression is associated with carcinogenesis. Elevated recombination frequency along with high-level expression of Rad51 was found in immortal human cell lines compared to mortal cells (Xia *et al*, 1997). Elevated expression of Rad51 enhances radioresistance of human tumour cells (Vispé *et al*, 1998; Yanagisawa *et al*, 1998). Overexpression of Rad51 protein in tumour cells renders them resistant against cytotoxic drugs like Cisplatin. Importantly, Ohnishi *et al* (1998) found that treatment of monolayer cultures of tumour cells with Rad51-specific antisense oligonucleotides renders those cells radiosensitive. Rad51 protein is highly expressed in tumour cell nuclei of several solid carcinomas (Maacke *et al*, 2000a). Rad51 protein interacts with a variety of tumour suppressor proteins including p53 (Stuerzbecher *et al*, 1996), BRCA1 (Scully *et al*, 1997) and BRCA2 (Sharan *et al*, 1997). Furthermore, Rad51 overexpression correlates with histological grading of the tumour in invasive ductal mammary carcinoma (Maacke *et al*, 2000b). Many reports demonstrate that Rad51 is not only involved in the progress of carcinogenesis but might also be highly significant in clinical practice (Henning and Stuerzbecher, 2003).

Up to now, there is no report concerning expression pattern of Rad51 in NSCLC and its clinical implication. Therefore, we used tissue microarrays containing resected specimens of 383 NSCLC patients for large-scale investigation of the prognostic value of Rad51 expression in NSCLC.

## MATERIALS AND METHODS

### Patients and clinical samples

We used primary tumour tissue samples from patients diagnosed with NSCLC pathological stage I–IV at the Lung Cancer Research Institute & Cancer Center of Guangdong province, P.R. China between January 1994 and December 1997. All patients were consecutive Chinese patients with NSCLC and underwent surgical treatment at the Department of Surgery of this institute. The study protocol was approved by the Ethical Committee of Guangdong Provincial People's Hospital, Guangdong province; P R. China and the Ethical Committee of University of Luebeck, Germany (file 03–153). Excluding criteria were as follows: (1) patients who had undergone chemotherapy or radiotherapy prior to surgery; (2) patients who died within 3 months after surgery; (3) patients whose cause of death remained unknown.

Totally, 383 patients and their corresponding resected tumour specimens were recruited in this study. These patients had a median follow-up of 34 months (range from 4 to 106 months). The last follow-up was carried out in January of 2003. All detailed information about demography, clinical manifestation and histopathology of the patients was collected retrospectively. In many cases of morphologically anaplastic large-cell carcinoma, assignment to squamous cell carcinoma (SCC) or adenocarcinoma (ADC) was verified by immunohistochemical demonstration of the cytokeratin profile.

All surgically resected tumour specimens and control specimens were fixed with 10% formalin and embedded in paraffin and obtained from the archives of our institute. The tumours were staged according to the International Union against Cancer's tumour-node-metastasis (TNM) classification (Mountain, 1997) and histologically subtyped and graded according to the World Health Organization guidelines (Sobin, 1982). All tumours and control tissues were reviewed by two pathologists (Dan Xie, Xin-yuan Guan).

### Microarray construction

Construction of NSCLC tissue microarray was as follows. Three most representative samples from each lung cancer case were adopted for the TMA construction. The individual donor tissue block and the corresponding histological haematoxylin–eosin-stained slides were overlaid for tissue TMA sampling. A TMA instrument (Beecher Instruments, Silver Spring, MD, USA) was used to create holes in a recipient paraffin block in order to obtain cylindrical core tissue biopsy samples with a diameter of 0.6 mm and to transfer these biopsy samples to the recipient block at defined array positions. Hence, three TMAs slides include a total of 1149 (383 × 3) samples. Multiple sections (5 *μ*m thick) were cut from the TMA block and mounted on microscope slides.

### Immunohistochemistry

Immunohistochemistry studies were performed using the standard avidin–biotin complex (ABC) method. In brief, TMA sections were deparaffinated and rehydrated. For antigen retrieval, TMA slides were immersed in antigen retrieval solution (10 mM citrate acid, pH 6) and boiled for 15 min in a pressure cooker. Endogenous peroxidase activity was blocked by incubation in 3.5% hydrogen peroxide in PBS for 15 min. After permeabilising the cells with Triton-X 100 for 5 min, specimens were blocked in horse serum and subsequently with avidin and biotin (Vector Laboratories, Burlingame, CA, USA). The TMA slides were incubated with monoclonal mouse anti-human Rad51 antibody 1G8 (1 mg ml^−1^; 1 : 5000 dilution) (Buchhop *et al*, 1996; Maacke *et al*, 2000b) for 60 min at 37°C in a moist chamber. After washing in PBS, the slides were then sequentially incubated with a biotinylated horse anti-mouse antibody (Vector Laboratories) at a concentration of 1 : 100 for 60 min at 37°C. The slides were then incubated with ABC complexes (Vector Laboratories) and stained using DAB (diaminobenzidine tetrahydrochloride; Vector Laboratories) as peroxidase substrate. Specimens were counterstained using Meyer's haematoxylin (Merck, Darmstadt, Germany). The slides were dehydrated and placed on coverslips. Negative control was performed by replacing the primary antibody with blocking serum. Slides from specimens that scored positive in previous experiments were used as positive controls.

### Assessment of immunohistochemistry

The immunostaining of TMA slides were assessed by two pathologists (Dan Xie, Xin-yuan Guan) without being aware of the clinical, pathological and follow-up data. The concept of positive-cell index (PCI) (positive-stained cell index), which means the proportion of positively stained tumour cells, was adapted for the analyses in this study. At least 200 tumour cells were counted to calculate the PCI in each specimen on TMA.

Since this study was focused on the prognostic role of molecular tumour markers, an optimal cutoff point of marker index was determined that allowed best separation of patients into those with favourable and those with unfavourable clinical features and prognosis. For this procedure, the corresponding *P*-values for all candidates of cutoff points (numerical percentage values) were calculated (Kronqvist *et al*, 1998) and the cutoff point representing the highest statistical significance (i.e. the lowest *P*-value) was regarded as the optimal threshold to separate prognostically different groups. Using this method, a PCI of 10% was identified as the optimal cutoff. Cases whose IHC scores were ⩽10% were called ‘low-level expressors’, whereas those with IHC scores >10% were defined as ‘high-level expressors’.

### Statistical analysis

All statistical analyses were performed using the SPSS 10.0. statistical software package (SPSS Inc., Chicago, IL, USA). Correlations between Rad51 expression and given categorised parameters were evaluated using the nonparametric Mann–Whitney *U*-test (for two categories) or Kruskal–Wallis test (for multiple categories). Kaplan–Meier curves were plotted from overall survival data, and the log-rank test was used for analysis of differences between patients with different level of Rad51 expression.

Patients, who were still alive or lost to follow-up or died of other causes than lung cancer recurrence or metastasis before January 2003, were treated as censored data in the survival analyses. Multivariate survival analysis was performed using the Cox Proportional Regression Hazard Model. For multivariate analysis, all variables that proved to be significant in the univariate analysis were selected in a stepwise fashion (forward selection of covariates) to evaluate the predictive power of each variable independently of the others. A *P*-value of 0.1 was adopted as the limit for entering and removing covariates. The relative risks (RR) and the associated 95% confidence intervals were calculated for the prognostic factors that contributed significantly to the model. For all analyses, statistical significance was assumed at a *P*-level of <0.05.

## RESULTS

### Clinicopathologic variables and clinical outcome

A total of 383 patients suffering from NSCLC were included in this study. Demographic, clinical and histopathological data (including age, sex, histology, TNM stage, surgical procedure, tumour grade and primary tumour site) were collected retrospectively. Data on smoking history were missing for the majority of patients.

The median age of the patients was 59 years (range, 22–94 years) at the time of diagnosis, 71.3% of the patients were male. In the majority of cases, lobectomy was performed (301 of 383 patients); 54 patients underwent pneumonectomy; 28 patients received conservative lung resection. Complete resection was done for 343 patients, incomplete resection for 40 patients. Incomplete resection means either macroscopic evidence of the tumour or metastatic lymph nodes left behind, microscopic evidence of the tumour on the resected stump or clinical evidence of a distant metastasis.

Histopathological examination of resected tumours revealed that ADC was the dominant histological group (192 cases), 132 cases were SCCs, 44 adenoSCCs, two anaplastic large-cell carcinomas, five lung sarcomas and eight were other less common malignant neoplasms including carcinoid tumour, adenoid cystic carcinoma and mucoepidermoid carcinoma.

Postoperative staging evaluation demonstrated that 110 had stage I disease, 91 had stage II disease, 124 had stage IIIA disease, 37 had stage IIIB disease, and 21 had stage IV disease. In all, 21 had T_1_ disease, 194 had T_2_ disease, 130 had T_3_ disease and 38 had T_4_ disease. A total of 171 had N_0_ disease, 78 had N_1_ disease, 126 had N_2_ disease and eight had N_3_ disease. In total, 209 tumours were located in the upper lobe, 31 were in the middle lobe, 130 were in the lower lobe and 13 were mixed or hard to be distinguished.

Univariate analyses of the demographic and clinical variables revealed that tumour state (T status), lymph node status (N status), metastasis status (M status), clinical stage and tumour grade all had prognostic significance with regard to overall survival. The results are presented in Table 1.

### Pattern of Rad51 expression in NSCLC and correlation to clinicopathological parameters

Immunochemical staining revealed 340 informative cases that could be assessed and scored under the light microscope. Positive tumour cells generally showed a nuclear staining pattern in NSCLC. There were much more positive nuclear staining cells in tumour tissue compared to normal lung tissue. This pattern allowed the classification of Rad51 expression by PCI. The expression status of Rad51 was classified into two groups: (1) low-level expression: PCI⩽10%; (2) high-level expression: PCI >10%.

**Figure 1:**
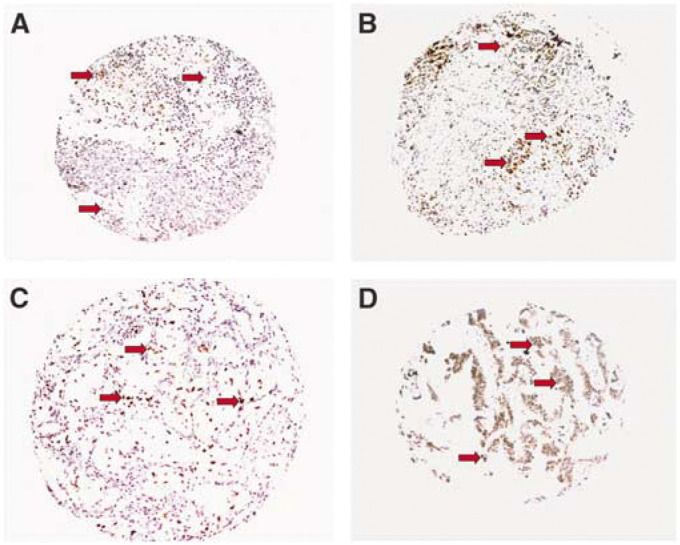
Representative examples of Rad51 protein expression in NSCLC. (**A**) Low-level expression of Rad51 in SCC (PCI<10%); (**B**) high-level expression of SCC (PCI=60%); (**C**) low-level expression of ADC (PCI<10%); (**D**) high-level expression of ADC (PCI=80%); magnification 1 × 10; detection of Rad51 protein by immunohistochemistry using the monoclonal anti-Rad51 antibody 1G8 (Buchhop *et al*, 1996); counterstaining with Hemalum; red arrows mark tumour cells expressing high levels of Rad51.

**Figure 2:**
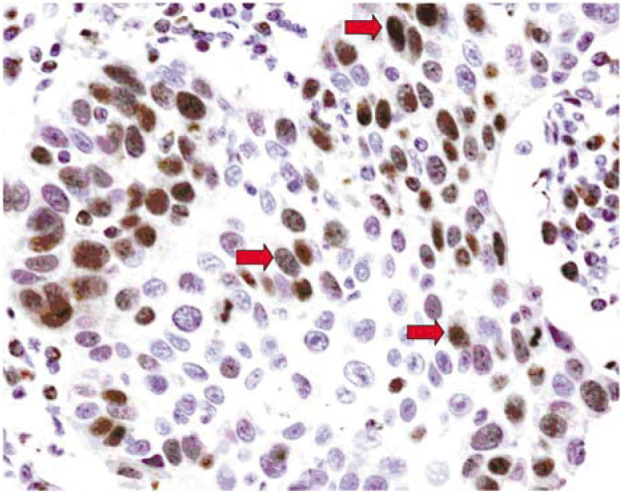
Representative example of high-level expression of Rad51 in lung squamous cell lung carcinoma at magnification 10 × 40. Detection of Rad51 protein by immunohistochemistry using the monoclonal anti-Rad51 antibody 1G8 (Buchhop *et al*, 1996); counterstaining with Hemalum; red arrows mark tumour cells expressing high levels of Rad51.

Figure 1 illustrates representative examples of the different levels of Rad51 staining in SCC and ADC of NSCLC. Figure 2 gives the high order magnification picture of a SCC specimen exhibiting high-level expression of Rad51.

Rad51 expression status was correlated with clinicopathological features. The relationship between Rad51 expression and clinicopathological features in NSCLC is shown in Table 2. Of 340 (29.4%) NSCLC cases, 100 showed high-level expression of Rad51, including 32 of 117 (27.4%) SCC and 53 of 169 (31.4%) ADC.

We found no significant correlation between Rad51 expression and age, sex, tumour status (T status), lymph node status (N status), metastasis status (M status), clinical stage, tumour cell type and differentiation.

### Relationship between Rad51 expression and clinical outcome in NSCLC: univariate survival analysis

The results of Rad51 expression were analysed with regard to overall survival time of the patients. Univariate survival analysis (log-rank test) demonstrated significant association between overall survival and the status of Rad51 (*P*<0.0001). Patients with high-level expression of Rad51 had a poorer prognosis than did those with low-level expression. Expression of Rad51 had an important impact on lung cancer survival regardless of the histological cell type. Patients with high-level expression of Rad51 had a poorer prognostic outcome regardless of SCC (*P*<0.0001) and ADC (*P*=0.0423) of NSCLC. Additionally, survival analysis was performed with regard to Rad51 expression in subsets of patients with different clinical stages. The results demonstrated that high-level expression was a prognostic factor in stage I–III NSCLC patients (*P*-values: 0.0072, 0.0041 and 0.0016, respectively). However, Rad51 expression could not differentiate the outcome of stage IV NSCLC patients (*P*=0.6229).

**Figure 3:**
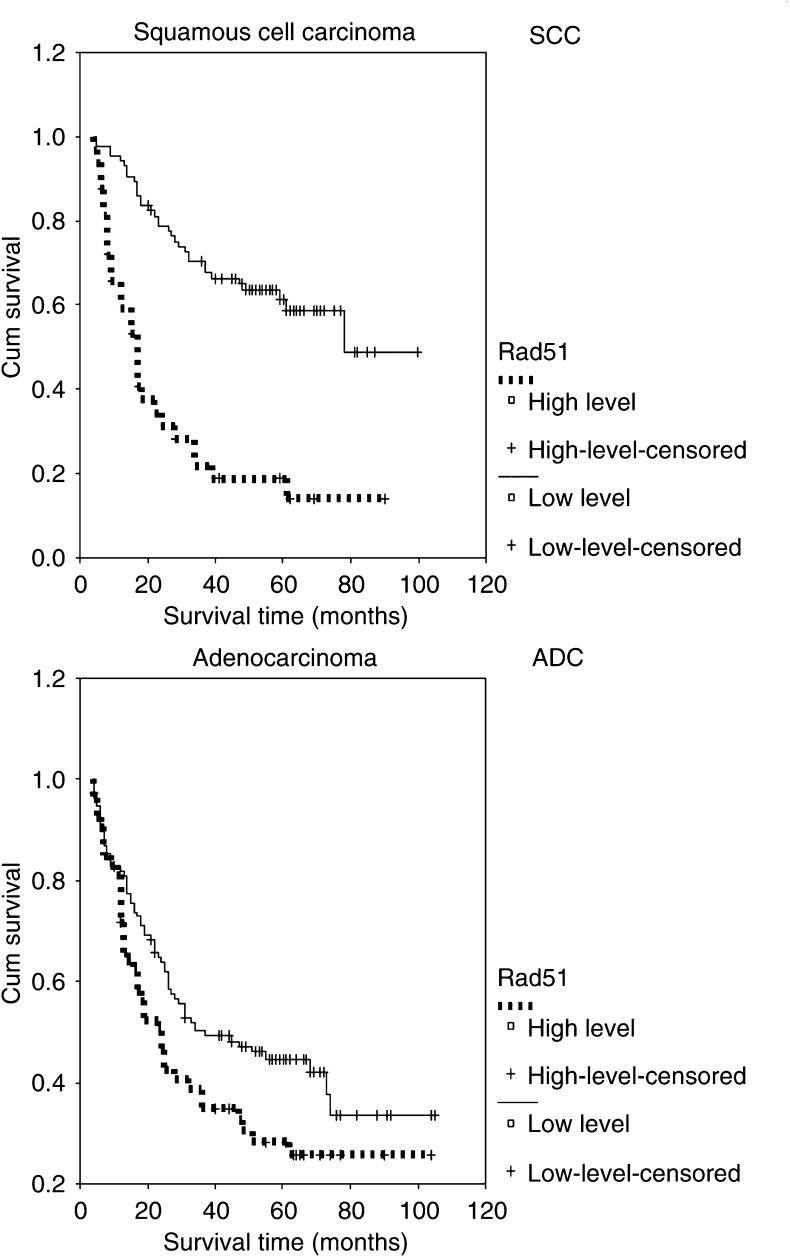
Kaplan–Meier survival analysis according to Rad51 expression in subsets of patients with SCC and ADC of the lung (log-rank test). SCC, probability of survival of squamous cell carcinoma of NSCLC patients: low-level expression (solid line), *n*=85; high-level expression (dashed line), *n*=32. ADC, probability of survival of adenocarcinoma of NSCLC patients: low-level expression (solid line), *n*=116; high-level expression (dashed line), *n*=53.

**Figure 4:**
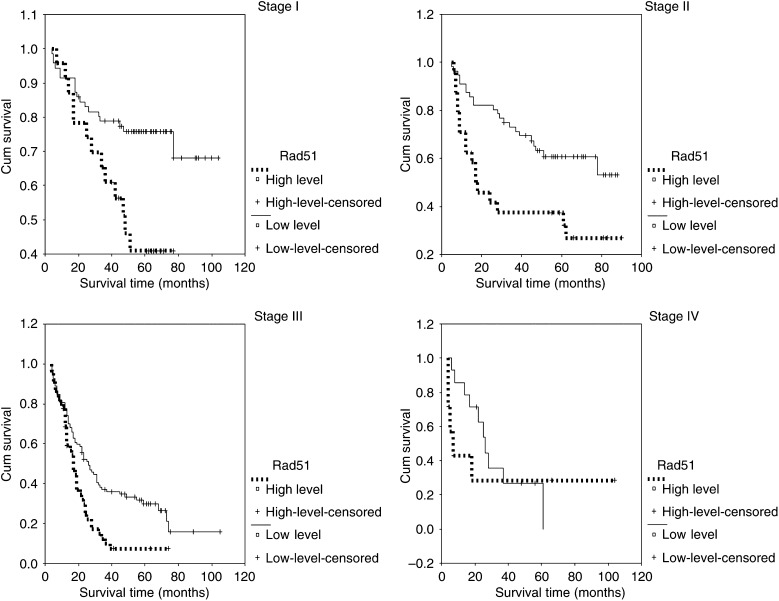
Kaplan–Meier survival analysis according to Rad51 expression in subsets of different clinical stage I–IV NSCLC patients (log-rank test): Stage I, probability of survival of stage I NSCLC patients: low-level expression (solid line), *n*=71; high-level expression (dashed line), *n*=24. Stage II, probability of survival of stage II NSCLC patients: low-level expression (solid line), *n*=56; high-level expression (dashed line), *n*=24. Stage III, probability of survival of stage III NSCLC patients: low-level expression (solid line), *n*=99; high-level expression (dashed line), *n*=45. Stage IV, probability of survival of stage IV NSCLC patients: low-level expression (solid line), *n*=14; high-level expression (dashed line), *n*=7.

Table 3 presents the results of univariate analysis with regard to Rad51 expression and overall survival of NSCLC. Figure 3 shows Kaplan–Meier survival curves in relation to Rad51 expression in subsets of patients with SCC and ADC of the lung. Figure 4 shows Kaplan–Meier survival curves in relation to Rad51 expression in subsets of different clinical stage NSCLC patients.

### Independent prognostic factors of NSCLC: multivariate Cox regression analysis

Since variables found to have prognostic influence by univariate analysis may covariate, all statistically significant variables from the univariate analysis were included in the multivariate regression analysis to identify independent prognostic factors. The data obtained are presented in Table 4. Rad51 expression proved to be an independent prognostic factor (*P*<0.001) as did lymph node metastasis (*P*=0.009), clinical stage (*P*=0.004) and tumour grade (*P*<0.001).

## DISCUSSION

At present, the TNM staging system is considered the most accurate predictor for NSCLC (Grondin and Liptay, 2002). However, the anatomic TNM staging system, based on histopathology and extent of disease at presentation, has reached its limit in providing critical information that may influence the strategy of treatment. Patients with the same pathological and clinical stage of NSCLC display considerable variability in recurrence and survival (Buccheri and Ferrigno, 1994). Therefore, a substantial amount of research on the biology of NSCLC has focused on evaluating the potential of new molecular markers present in tumour cells to serve as prognostic factors or possible targets for therapy. Although a variety of molecular markers have been implicated in the prognosis of NSCLC, conflicting results were widely reported in the literature (Brundage *et al*, 2002). Thus, further investigations are required to develop appropriate markers or panels of molecular markers, as well as good quality control in detection of the disease.

Rad51 is the key enzyme for homologous recombination, an evolutionarily conserved mechanism for the repair of DNA damage and the generation of genetic diversity (Henning and Stuerzbecher, 2003). In order to investigate the role of Rad51 in NSCLC, we examined immunohistochemically Rad51 expression in NSCLC tissues using high throughput tissue microarrays. Consistent with studies in several other tumour entities, including breast cancer and pancreatic ADC (Maacke *et al*, 2000a, 2000b; Han *et al*, 2002; Raderschall *et al*, 2002), our results demonstrate that Rad51 is overexpressed in tumour cell nuclei in NSCLC compared to normal tissues. In all, 29.4% of NSCLC showed high-level expression of Rad51. These findings, together with results from previous studies, suggest a role for abnormal expression of the mammalian Rad51 recombination protein in the multistep process of tumorigenesis.

Most importantly, this is the first study to evaluate the prognostic value of Rad51 in NSCLC patients. The data presented revealed that the median survival time and 5-year survival rate in patients with high-level expression of Rad51 appear shorter and lower than that of patients with low Rad51 expression. The median survival time of patients with Rad51 high-level expression was 19 months, compared to 68 months for patients with low-level expression. The 5-year survival rate of patients with Rad51 high-level expression was 24.9%, but for patients with low-level expression of Rad51 it was 50.3%. Additionally, stratified survival analysis by histological tumour cell type demonstrated that Rad51 expression was closely related to the survival of both lung SCC and lung ADC. Furthermore, Rad51 expression was an independent factor in NSCLC patients. Therefore, our findings demonstrate that Rad51 expression has the potential to predict the outcome of NSCLC patients. The assessment of Rad51 expression may, therefore, be used as an additional tool in identifying those patients at risk of tumour recurrence and progression, and it may be a helpful criterion to optimise individual therapy management.

These findings raise the question of the pathological role of Rad51 in the development of NSCLC. Elimination of Rad51 in mice leads to sensitivity towards ionising radiation and early embryonic lethality (Tsuzuki *et al*, 1996). Conditional inhibition of Rad51 transcription in untreated chicken DT40 cells results in G_2_/M arrest with high levels of chromosome-type breaks (Sonoda *et al*, 1998). These data suggest that Rad51 might be essential for survival in higher eukaryotic cells, presumably due to its role in repairing DNA double-strand breaks arising during DNA replication. Moreover, elevated expression of Rad51 and/or homologous recombination activity in immortal human cells may contribute to survival and/or increased genetic instability. Enhanced expression of Rad51 protein in tumour cells is associated with high DNA repair capacity, elevated recombination rates and increased resistance against radio- and chemotherapy (Henning and Stuerzbecher, 2003). Our present results of NSCLC and data from papillary bladder carcinoma (Krueger *et al*, unpublished), as well as breast cancer (Maacke *et al*, 2000b) and pancreatic cancer (Maacke *et al*, 2000a; Han *et al*, 2002) strongly suggest that high-level Rad51 expression might be a permissive event for tumour progression, probably because it helps to keep DNA damage at a tolerable level for cell survival and at the same time enhancing genetic instability. Unfortunately, no adequate information on adjuvant treatment of the patients was available in this retrospective series. Therefore, no conclusions can be drawn about a potential role of Rad51 expression as predictor of response to therapy. It is however tempting to speculate that Rad51 expression might also predict therapy response of the individual NSCLC patient on the basis of *in vitro* data, indicating the involvement of Rad51 activity in mechanisms of therapy resistance after the treatment of cells with DNA-damaging agents or irradiation (Henning and Stuerzbecher, 2003).

In summary, the present study introduces Rad51 expression as independent prognostic factor in NSCLC with elevated levels of Rad51 protein in tumour cells predicting poor outcome of the disease for the individual patient. Our findings have possible therapeutic applications. Rad51 could serve as a prognostic marker to improve tumour classification of NSCLC. More importantly, downregulation of Rad51 protein by Rad51 RNAi technology or Rad51-inhibitory drugs could be used to sensitise tumours to radiation or chemotherapy.

## Figures and Tables

**Table 1:** Predictive variables for prognosis of 383 NSCLC patients by univariate analysis (log-rank test)

**Variables**	**Patients (cases)**	**Mean survival (months)**	**Median survival (months)**	**5-year survival (%)**	***P*-value**
*Age*					
⩽59 years (median)	184	55	45	44	0.974
>59 years	199	55	37	44	
					
*Sex*					
Male	273	55	39	45	0.865
Female	110	55	34	42	
					
*T status*					
1	21	84	n.r.^a^	75	<0.001
2	194	61	49	47	
3	130	50	31	41	
4	38	29	17	21	
					
*N status*					
0	171	72	n.r.^a^	62	<0.001
1	78	57	45	48	
2	126	29	22	16	
3	8	21	16	0	
					
*M status*					
0	362	56	42	45	0.041
1	21	37	25	20	
					
*Stage*					
I	110	79	n.r.^a^	67	<0.001
II	91	59	62	54	
III	161	36	22	23	
IV	21	37	25	19	
					
*Histology*					
Squamous carcinoma	132	60	59	49	0.448
Adenocarcinoma	192	52	34	41	
Adenosquamous	44	47	33	41	
Others^b^	15	57	46	47	
					
*Tumour grade*					
G1	70	68	78	59	<0.001
G2	183	54	37	40	
G3	130	36	15	28	
					
*Surgical procedure*					
Lobectomy	301	57	45	46	0.222
Pneumonectomy	54	45	31	40	
Conservative resection^c^	28	37	32	26	

a n.r.=not reached.

b Others include anaplastic large-cell carcinoma, sarcoma, adenoid cystic carcinoma, mucoepidermoid carcinoma and carcinoid tumour.

c Conservative resections include segmentectomy and wedge resection.

**Table 2:** Relation between Rad51 expression and clinicopathological parameters in 235 cases of NSCL

	**Rad51 (cases and percentage)**		
**Parameters**	**−a**	**+^b^**	***P*-value**
*Total*	240 (70.6%)	100 (29.4%)	
*Age*			
⩽59 years	119 (73.5%)	43 (26.5%)	0.269
>59 years	121 (68.0%)	57 (32.0%)	
			
*Sex*			
Male	171 (70.1%)	73 (29.9%)	0.744
Female	69 (71.9%)	27 (28.1%)	
			
*T status*			
1	14 (66.7%)	7 (33.3%)	0.771
2	124 (72.9%)	46 (27.1%)	
3	77 (67.5%)	37 (32.5%)	
4	25 (71.4%)	10 (28.6%)	
			
*N status*			
0	105 (70.5%)	44 (29.5%)	0.513
1	54 (77.1%)	16 (22.9%)	
2	76 (67.3%)	37 (37.2%)	
3	5 (62.5%)	3 (37.5%)	
			
*M status*			
0	226 (70.8%)	93 (29.2%)	0.684
1	14 (66.7%)	7 (33.3%)	
			
*Stage*			
I	71 (74.7%)	24 (25.3%)	0.756
II	56 (70.0%)	24 (30.0%)	
III	99 (68.8%)	45 (31.3%)	
IV	14 (66.7%)	7 (33.3%)	
			
*Histology*			
SCC^c^	85 (72.6%)	32 (27.4%)	0.891
ADC^d^	116 (68.6%)	53 (31.4%)	
ADSCC^e^	29 (72.5%)	11 (27.5%)	
Others^f^	10 (71.4%)	4 (28.6%)	
			
*Tumour grade*			
G1	41 (66.1%)	21 (33.9%)	0.535
G2	109 (66.7%)	52 (33.3%)	
G3	90 (76.9%)	27 (23.1%)	

a −=low-level expression of Rad51.

b +=high-level expression of Rad51.

c SCC=squamous cell carcinoma.

d ADC=adenocarcinoma.

e ADSCC=adenosquamous carcinoma.

f others include anaplastic large-cell carcinoma, sarcoma, adenoid cystic carcinoma, mucoepidermoid carcinoma and carcinoid tumour.

**Table 3:** Prognostic value of Rad51 in NSCLC patients by univariate analysis (log-rank test)

**Rad51 expression**	**Patients (cases)**	**Mean survival (months)**	**Median survival (months)**	**5-year survival (%)**	***P*-value**
*Total*					
Low-level expression	240	61	68	50.3	<0.0001
High-level expression	100	38	19	24.9	
					
*Cell type*					
Squamous carcinoma					
Low-level expression	85	68	78	61.3	<0.0001
High-level expression	32	28	17	18.8	
					
Adenocarcinoma					
Low-level expression	116	54	37	44.8	0.0423
High-level expression	53	42	24	28.3	
					
*Clinical stage*					
Stage I					
Low-level expression	71	82	n.r.^a^	75.7	0.0072
High-level expression	24	49	48	40.9	
					
Stage II					
Low-level expression	56	63	n.r.^a^	60.8	0.0041
High-level expression	24	39	17	37.5	
					
Stage III					
Low-level expression	99	41	26	30.0	0.0016
High-level expression	45	21	17	7.4	
					
Stage IV					
Low-level expression	14	32	26	26.8	0.6229
High-level expression	7	35	7	28.6	

a n.r.=not reached.

**Table 4:** Multivariate analysis on overall survival (Cox regression model)

**Factors**	**Relative risk (RR)**	**95% CI**	***P*-value**
N status^a^	1.368	1.080–1.733	0.009
Stage^b^	1.433	1.123–1.829	0.004
Differentiation^c^	0.608	0.494–0.747	<0.001
Rad51^d^	1.926	1.435–2.585	<0.001

a N_3_ *vs* N_2_ *vs* N_1_ *vs* N_0_.

b I *vs* II *vs* III *vs* IV.

c Poor *vs* moderate *vs* well.

d High-level expression *vs* low-level expression.
